# Transportation of *Aspergillus nidulans* Class III and V Chitin Synthases to the Hyphal Tips Depends on Conventional Kinesin

**DOI:** 10.1371/journal.pone.0125937

**Published:** 2015-05-08

**Authors:** Norio Takeshita, Valentin Wernet, Makusu Tsuizaki, Nathalie Grün, Hiro-omi Hoshi, Akinori Ohta, Reinhard Fischer, Hiroyuki Horiuchi

**Affiliations:** 1 Department of Microbiology, Institute for Applied Biosciences, Karlsruhe Institute of Technology (KIT), Karlsruhe, Germany; 2 Department of Biotechnology, The University of Tokyo, Bunkyo-ku, Tokyo, Japan; 3 Faculty of Life and Environmental Sciences, University of Tsukuba, Tsukuba, Ibaraki, Japan; Cinvestav, MEXICO

## Abstract

Cell wall formation and maintenance are crucial for hyphal morphogenesis. In many filamentous fungi, chitin is one of the main structural components of the cell wall. *Aspergillus nidulans* ChsB, a chitin synthase, and CsmA, a chitin synthase with a myosin motor-like domain (MMD) at its N-terminus, both localize predominantly at the hyphal tip regions and at forming septa. ChsB and CsmA play crucial roles in polarized hyphal growth in *A*. *nidulans*. In this study, we investigated the mechanism by which CsmA and ChsB accumulate at the hyphal tip in living hyphae. Deletion of *kinA*, a gene encoding conventional kinesin (kinesin-1), impaired the localization of GFP-CsmA and GFP-ChsB at the hyphal tips. The transport frequency of GFP-CsmA and GFP-ChsB in both anterograde and retrograde direction appeared lower in the *kinA*-deletion strain compared to wild type, although the velocities of the movements were comparable. Co-localization of GFP-ChsB and GFP-CsmA with mRFP1-KinA^rigor^, a KinA mutant that binds to microtubules but does not move along them, was observed in the posterior of the hyphal tip regions. KinA co-immunoprecipitated with ChsB and CsmA. Co-localization and association of CsmA with KinA did not depend on the MMD. These findings indicate that ChsB and CsmA are transported along microtubules to the subapical region by KinA.

## Introduction

Hyphae of filamentous fungi represent extremely polarized cells. Since “polarized growth” is a general growth mechanism common in the elongation of cells as distinct as neurons or pollen tubes, as well as in the differentiation of embryos [1], the mechanism of hyphal tip growth in filamentous fungi may provide a helpful basic model. This mechanism is thought to be highly elaborate and is supported by the continuous delivery of vesicles containing proteins required for polarized growth to the hyphal tips [2,3]. In some filamentous fungi, vesicles delivered to hyphal tips accumulate at apices prior to fusion with the membrane. These clusters of vesicles are called Spitzenkörper or apical bodies [4]. The Spitzenkörper is thought to act as a vesicle supply center, the place where cargo for the hyphal apex is sorted [5]. In the ascomycete filamentous fungus *Aspergillus nidulans*, some proteins involved in polarized growth are transported along microtubules and the actin cytoskeleton to the hyphal tips [6–9]. While there are 5~8 linear microtubules that extend to the hyphal tips in *A*. *nidulans*, actin cables are formed preferentially at the apical hyphal regions. The vesicles containing components of the tip growth machineries are thought to be transported along microtubules from posterior parts of hyphal tip regions to their apical regions, transferred to actin cables and delivered to the apical cortices of hyphae [10–12].

The cell surface of filamentous fungi is covered with a cell wall that consists mainly of chitin and glucan. Chitin is a β-1,4-linked homopolymer of *N*-acetylglucosamine and its synthesis is important for hyphal morphogenesis [13]. To date, fungal chitin synthases have been classified into seven classes and three divisions according to their structural characteristics [14]. In this report, we follow the classification proposed by Chigira et al. and Choquer et al. [15,16]. Fungal chitin synthases are membrane-bound proteins containing several transmembrane regions. Class I, II, and III chitin synthases belong to division I and class IV, V, and VI belong to division II. We previously isolated six chitin synthase genes from *A*. *nidulans*: *chsA*, *chsB*, *chsC*, *chsD*, *csmA*, and *csmB*; the products of these genes belong to classes II, III, I, IV, V, and VI, respectively [17–21](S1 Fig). The class V and VI chitin synthases, CsmA and CsmB, respectively, consist of a C-terminal chitin synthase domain (CSD) containing the amino acid sequences conserved in chitin synthases of other classes [22], and an N-terminal myosin motor-like domain (MMD). Myosins are motor proteins that move along actin filaments. In our previous studies, both *csmA-* and *csmB*-deletion mutations caused similar phenotypic abnormalities (e.g., formation of balloons and intrahyphal hyphae, and hyphal lysis especially under low osmotic conditions) [20,23], and these mutations were synthetically lethal [20]. The *chsB*-deletion mutant formed extremely small colonies and did not form conidiospores [24]. CsmA, CsmB, and ChsB localized near actin structures at the hyphal tips and at forming septa [20,24,25]. The MMDs of CsmA and CsmB bound to actin filaments *in vitro* [20,25] and the interaction between each MMD and actin was crucial for their proper localization and function [25,26]. However, the MMD of CsmB is not functionally equivalent to that of CsmA [27].

The orthologous genes encoding classes III, V, and VI chitin synthases have been isolated from filamentous fungi and some dimorphic yeasts, and their functions have been investigated [28–39]. The results obtained from these investigations indicate that the chitin synthases belonging to classes III, V, and VI play important roles in hyphal tip growth, maintenance of cell wall integrity, and pathogenicity.

Since the genomes of the yeasts *Saccharomyces cerevisiae*, *Schizosaccharomyces pombe*, and a dimorphic yeast *Candida albicans* do not possess classes III, V, and VI chitin synthases, the chitin synthases in these classes likely play critical roles in polarized growth, especially in filamentous fungi. In order to understand the dynamic aspects of polarized hyphal growth, it is necessary to clarify their transport and localization mechanisms in the hyphae.

Kinesins are motor proteins that move on microtubules in the plus end direction and are divided into 15 families according to their structural properties [40]. In these families, kinesin-1, kinesin-3, and kinesin-7 are involved in polarized growth of filamentous fungi [41]. In *A*. *nidulans*, there are 11 kinesin-encoding genes [42]. Among these genes, *kinA*, *uncA*, and *kipA* encode kinesins belonging to kinesin-1, kinesin-3, and kinesin-7, respectively. Kinesin-1 is suggested to function in the transport of secretory vesicles, dyneins, and dynactin, a microtubule minus end-directed motor and its regulator, while Kinesin-3 is suggested to be involved in the transport of secretory vesicles, early endosomes, peroxisomes, and mRNPs containing mRNA [11,43–53]. Zhang et al. [53] and Yao et al. [51] showed that dynein and dynactin localize along microtubules in the Δ*kinA* mutant, which provide good support for the idea that KinA transports dynein/dynactin along microtubules to the plus ends.

Although the localization of chitin synthases has already been investigated in some filamentous fungi, their localization mechanisms remain largely unknown. In *N*. *crassa* chitin synthases are thought to be transported on special vesicles called chitosomes [54,55]. In basidiomycete dimorphic yeast *Ustilago maydis*, it was reported that the proper localization of class V chitin synthase Msc1 depends on the presence of microtubules and/or actins, that its transport along microtubules does not depend on its MMD, and that the MMD is required for the tethering of Mcs1 at the hyphal tips to increase the efficiency of its exocytosis [36,50]. In this study, we report the transporting mechanisms of classes III and V chitin synthases, ChsB and CsmA, to hyphal tips in *A*. *nidulans* and the role of the MMD of CsmA in the transport process.

## Materials and Methods

### Strains, media, and bacterial and fungal transformations

The *A*. *nidulans* strains used in this study are listed in Table 1. Complete medium, YGuu medium (0.5% yeast extract, 1% glucose, 0.1% trace elements, uracil at 1.12 mg/ml, and uridine at 2.44 mg/ml) and minimal medium (MMGuu), minimal medium containing 2% glycerol (MMGlyuu), or minimal medium containing 100 mM threonine and 0.1% fructose instead of glucose (MMTFuu) for *A*. *nidulans* were used [56,57]. YGuu, MMGuu, and MMTFuu plates contained 1.5% agar. MMGuu and MMTFuu were supplemented with arginine at 0.2 μg/ml, biotin at 0.02 μg/ml, *p*-aminobenzoic acid at 1 μg/ml, and pyridoxine at 0.5 μg/ml, when necessary. Bacterial and fungal transformations were performed as described previously [57–59].

**Table 1:** *A*. *nidulans* strains used in this study.

Strain	Genotype	Source
ABPU1	*biA1 pyrG89 argB2 pyroA4 wA3*	[19]
A1149	*pyrG89 pyroA4* Δ*nkuA*::*argB*	FGSC*
CsmA-HA/FLAG-ChsB1, 2	*biA1 pyrG89 argB2 pyroA4 wA3* Δ*csmA*::*csmA*-*9xHA-pyrG* Δ*argB*::*argB*-*chsB*(p)-*3xFLAG*-*chsB*	This study
EGFP-CsmA1, 2	*biA1 pyrG89 argB2 pyroA4 wA3* Δ*csmA*::*argB*-*alcA*(p)-*egfp*-*csmA*	This study
EB-5	*biA1 pyrG89 argB2 pyroA4 wA3* Δ*chsB*::*pyr4*-*alcA*(p)-*chsB* Δ*argB*::*argB*-*chsB*(p)-*egfp*-*chsB*	[24]
SNR1	Δ*argB*::*trp*Δ*B* Δ*kinA*::*pyr4 pyrG89 pyroA4 yA2*	[45]
SNZ9	*pyrG89 argB2* Δ*nkuA*::*argB pyroA4* Δ*uncA*::*pyroA*	[48]
EGFP-CsmAΔkinA1, 2	Δ*argB*::*trpC*Δ*B* Δ*kinA*::*pyr4 pyrG89 pyroA4 yA2* Δ*csmA*::*argB*-*alcA*(p)-*egfp*-*csmA*	This study
EGFP-CsmAΔuncA1, 2	*biA1 pyrG89 argB2 pyroA4 wA3* Δ*csmA*::*argB*-*alcA*(p)-*egfp*-*csmA* Δ*uncA*::*pyroA*	This study
EGFP-ChsBΔkinA1, 2	Δ*argB*::*trpC*Δ*B* Δ*kinA*::*pyr4 pyrG89 pyroA4 yA2* Δ*argB*::*argB*-*chsB*(p)-*egfp*-*chsB*	This study
EGFP-ChsBΔuncA1, 2	*argB2 pyroA4 wA3* Δ*argB*::*argB*-*chsB*(p)-*egfp*-*chsB* Δ*uncA*::*pyroA*	This study
EGFP-CsmA/mRFP-KinA^rigor^1, 2	*biA1 pyrG89 argB2 pyroA4 wA3* Δ*csmA*::*argB*-*alcA*(p)-*egfp*-*csmA* Δ*kinA*::*pyroA*-*alcA*(p)-*mrfp*-*kinA* ^*rigor*^	This study
EGFP-ChsB/mRFP-KinA^rigor^1, 2	*biA1 pyrG89 argB2 pyroA4 wA3* Δ*chsB*::*pyr4*-*alcA*(p)-*chsB* Δ*argB*::*argB*-*chsB*(p)-*egfp*-*chsB* Δ*kinA*::*pyroA*-*alcA*(p)-*mrfp*-*kinA* ^*rigor*^	This study
ABPU1/mRFP-KinA^rigor^1, 2	*biA1 pyrG89 argB2 pyroA4 wA3* Δ*kinA*::*pyroA*-*alcA*(p)-*mrfp*-*kinA* ^*rigor*^	This study
CA2	*biA1 pyrG89 argB2 pyroA4 wA3* Δ*csmA*::*csmA*-*9xHA-pyrG*	[61]
FB-3	*biA1 pyrG89 argB2 pyroA4 wA3* Δ*chsB*::*pyr4*-*alcA*(p)-*chsB* Δ*argB*::*argB*-*chsB*(p)-*3XFLAG*-*chsB*	[24]
CsmA-HA/mRFP-KinA^rigor^1, 2	*biA1 pyrG89 argB2 pyroA4 wA3* Δ*csmA*::*csmA*-*9HA-pyrG* Δ*kinA*::*pyroA*-*alcA*(p)-*mrfp*-*kinA* ^*rigor*^	This study
FLAG-ChsB/ mRFP-KinA^rigor^1, 2	*biA1 pyrG89 argB2 pyroA4 wA3* Δ*chsB*::*pyr4*-*alcA*(p)-*chsB* Δ*argB*::*argB*-*chsB*(p)-*3XFLAG*-*chsB* Δ*kinA*::*pyroA*-*alcA*(p)-*mrfp*-*kinA* ^*rigor*^	This study
EGFP-ΔMA1, 2	*biA1 pyrG89 argB2 pyroA4 wA3* Δ*csmA*::*argB*-*alcA*(p)-*egfp*-*csmA*(Δ1–860)	This study
EGFP-ΔMA/ mRFP-KinA^rigor^1, 2	*biA1 pyrG89 argB2 pyroA4 wA3* Δ*csmA*::*argB-alcA*(p)-*egfp*-*csmA*(Δ1–860) Δ*kinA*::*pyroA*-*alcA*(p)-*mrfp*-*kinA* ^*rigor*^	This study
CΔMHA	*biA1 pyrG89 argB2 pyroA4 wA3* Δ*csmA*::*argB*-*alcA*(p)*-csmA*(Δ1–860)-*9xHA-pyrG*	[25]
D10HA	*biA1 pyrG89 argB2 pyroA4 wA3* Δ*csmA*::*argB-alcA*(p)*-csmA*(Δ347–356)-*9xHA-pyrG*	[25]
CΔMHA/ mRFP-KinA^rigor^1, 2	*biA1 pyrG89 argB2 pyroA4 wA3* Δ*csmA*::*argB-alcA*(p)-*csmA*(Δ1–860)-*9xHA-pyrG* Δ*kinA*::*pyroA*-*alcA*(p)-*mrfp*-*kinA* ^*rigor*^	This study
D10HA/ mRFP-KinA^rigor^1, 2	*biA1 pyrG89 argB2 pyroA4 wA3* Δ*csmA*::*argB-alcA*(p)-*csmA*(Δ347–356)-*9xHA-pyrG* Δ*kinA*::*pyroA*-*alcA*(p)-*mrfp*-*kinA* ^*rigor*^	This study
ABPU1/GFP-KinA1, 2	*biA1 pyrG89 argB2 pyroA4 wA3* Δ*kinA*::*pyroA-alcA*(p)*-gfp-kinA*	This study
CsmA-HA/GFP-KinA1, 2	*biA1 pyrG89 argB2 pyroA4 wA3* Δ*kinA*::*pyroA-alcA*(p)*-gfp-kinA* Δ*csmA*::*pyrG*-*csmA*-*9HA*	This study
CsmA-HA/FLAG-ChsB/ mRFP-KinArigor1, 2	*biA1 pyrG89 argB2 pyroA4 wA3* Δ*csmA*::*csmA-9xHA-pyrG argB*::*chsB(p)-3xFLAG-chsB* Δ*kinA*::*pyroA4-alcA(p)-mrfp-kinA* ^*rigor*^	This study

*: Fungal Genetics Stock Center, Kansas City, Missouri

### Constructions of plasmids and *A*. *nidulans* strains

To create the strains that produced CsmA with an EGFP tag at its N-terminus, we constructed the plasmid pMAEC as follows: The 0.8-kb *Kpn*I-*Pst*I fragment of pM-ALC2 [25] was cloned into the *Kpn*I-*Pst*I site of pUC19, yielding pUCCSM2. The 0.7-kb fragment encoding EGFP was amplified by PCR from pEGFP (Clontech) using primers 5′-GCGTTAACATGGTGAGCAAGGGCGAGG-3′ and 5′-GCCCATGGTCTTGTACAGCTCGTCCAT-3′, in which the underlined letters represent *Hinc*II and *Nco*I recognition sites, respectively, digested with *Hinc*II and *Nco*I, and cloned into the *Nco*I site of pUCCSM2, yielding pUCCSM. The 1.5-kb *Kpn*I-*Pst*I fragment of pUCCSM was cloned into the *Kpn*I-*Pst*I sites of pM-ALC2, yielding pMAEC. The 4.1-kb *Eco*RI and *Pst*I fragment of pMAEC was used for the transformation of the ABPU1 and SNR1 (Δ*kinA*) strains (Table 1). Two transformants obtained from each strain, in which the DNA fragment encoding EGFP was integrated into the 5′-region of the *csmA* cording region, were selected and confirmed by Southern blot analysis using the 1.0-kb *Spe*I-*Xho*I fragment of pM-ALK-CHS5 [23] as a probe, and were designated as EGFP-CsmA1 and 2, and EGFP-CsmAΔkinA1 and 2, respectively (Table 1).

To obtain the strains that produced EGFP-CsmA in an *uncA* deletion mutant, a 4.0-kb fragment amplified from pNZ13 [48] using primers UncA-LB-fwd (5′-CGTCGATGGAAGGCATATACTACTCGC-3′) and UncA-RB-rev (5′-CATCCACGTCCCCATAACTAATACCACC-3′) was used for the transformation of EGFP-CsmA1. Two transformants in which *uncA* was replaced with *pyroA* were selected and designated as EGFP-CsmAΔuncA1 and 2.

EB-5 (*chsB*(p)_*-*_ *egfp-chsB*) [24] was crossed with SNZ9 (Δ*uncA*) [48], selected and designated as EGFP-ChsBΔuncA1 and 2. SNR1 (Δ*kinA*) was transformed with the *Bgl*II fragment of pEB-argB [24]. Two transformants in which *chsB*(p)*-egfp-chsB* was integrated into the *argB* locus were selected and designated as EGFP-ChsBΔkinA1 and 2.

The strains that produced CsmA without its MMD (ΔMA) tagged with EGFP at its N-terminus under the control of the *alcA* promoter were constructed as follows: The 4.2-kb fragment containing the *csmA* promoter, *argB*, the *alcA* promoter, and the coding region of EGFP was amplified with AP1 (5′-ATAGTAACAGGTCAGGGTAT-3′) and 3-DMA-EGFP (5′-AGTTGTGAAACATATCGCCCCTTGTACAGCTCGTCCATGC-3′) as primers using pMAEC as a template and the 1.0-kb fragment encoding the CSD of CsmA (861 a.a.) was amplified from pMAEC using 5-EGFP-DMA (5′-GCATGGACGAGCTGTACAAGGGGCGATATGTTTCACAACT-3′) and 3-DMA (5′-CACATGGCCGACAATGAACA-3′) as primers. The amplified 4.2-kb and 1.0-kb fragments were fused by double-joint PCR [60] and the obtained fragment designated as EGFP-ΔMA was used for transformation. Two transformants in which a single copy of the EGFP-ΔMA producing fragment was integrated into the *csmA* locus were selected by Southern blot analysis using the same strategy as in the case of EGFP-CsmA1, and were designated EGFP-ΔMA1 and 2. The strains that produced KinA^rigor^ tagged with mRFP at their N-termini were obtained as follows: EGFP-CsmA1, EB-5, ABPU1, CA2, FB-3 [24,61], EGFP-ΔMA1, CΔMHA, and D10HA [25] were transformed with the 5.1-kb *Eco*RI-*Bgl*II fragment of pCS5-NZ [48]. The transformants in which the wild-type *kinA* was replaced with *alcA*(p)*-mrfp-kinA* ^*rigor*^ were selected and designated as EGFP-CsmA/mRFP-KinA^rigor^1 and 2, EGFP-ChsB/mRFP-KinA^rigor^1 and 2, ABPU1/mRFP-KinA^rigor^1 and 2, CsmA-HA/mRFP-KinA^rigor^1 and 2, FLAG-ChsB/mRFPKinA^rigor^1 and 2, EGFP-ΔMA/mRFP-KinA^rigor^1 and 2, CΔMHA/mRFP-KinA^rigor^1 and 2, D10HA/mRFP-KinA^rigor^1 and 2, respectively (Table 1). The strains that produced CsmA tagged with 9xHA at its C-terminus and ChsB tagged with 3xFLAG at its N-terminus were obtained by the transformation of CA2 with pFB-argB [24] and were designated as CsmA-HA/FLAG-ChsB1 and 2 (Table 1). CsmA-HA/FLAG-ChsB1 was transformed with the 5.1-kb *Eco*RI-*Bgl*II fragment, and the transformants in which the wild-type *kinA* was replaced with *alcA*(p)*-mrfp-kinA* ^*rigor*^ were designated as CsmA-HA/FLAG-ChsB/mRFPKinA^rigor^1 and 2. To obtain the strain that produce KinA tagged with GFP at its N-terminus, the ABPU1 strain was transformed with the 5.1-kb *Eco*RI-*Bgl*II fragment of pCS2-NZ [48]. Two transformants in which the transformed fragment was integrated into the *kinA* locus were selected and designated as ABPU1/GFP-KinA1 and 2. ABPU1/GFP-KinA1 was transformed with the 3.0-kb *Bgl*II-*Eco*RI fragment of pHA9 [61]. Two transformants in which the fragment was integrated into the *csmA* locus were selected and designated as CsmA-HA/GFP-KinA1 and 2.

We obtained at least two transformants in each case after the confirmation of their relevant DNA structures by Southern blot analysis. They exhibited the same phenotypes with respect to growth and hyphal morphology. We used one of them for further experiments.

### Light/fluorescence microscopy

Live-cell imaging of germlings and young hyphae: cells were grown on cover slips in 0.5 ml MMGly (de-repression of the *alcA* promoter) were used. Although GFP-ChsB was expressed under the native promoter, to compare the data of GFP-CsmA, which was expressed under the *alcA* promoter, MMGly was selected. Cells were incubated at 30°C overnight or 1 day. The cover slips were mounted on a microscope slide. Tempcontrol mini (Pepcon) was used as needed to control the temperature of the specimens during microscopy. Images were captured using an Axiophot microscope using a Planapochromatic 63 times oil immersion objective lens, the Zeiss AxioCam MRM camera (Zeiss, Jena, Germany), and the HBO103 mercury arc lamp (Osram) or HXP 120 (Zeiss, Jena, Germany) possessing faster speed wavelength switching. Images were collected and analyzed using the AxioVision and Zen system (Zeiss). Kymographs were made using ImageJ software (http://rsb.info.nih.gov/ij/) and Zen system (Zeiss).

### Other methods

Western blot analysis was performed as described previously [62] with slight modifications. Cell lysates were prepared as follows: Conidia were inoculated in MMTFuu liquid medium and incubated for 14–16 h. The mycelia were frozen with liquid nitrogen and broken by grinding. The cell lysates were fractionated as described previously [24,61]. 10,000 x g pellet (P10) and the 100,000 x g pellet (P100) were suspended with 100 μl of TNE buffer (20 mM Tris-HCl pH 7.4, 150 mM NaCl, 2 mM EDTA) and then 500 μl of IP buffer (0.1% Triton X-100, 100 mM NaCl, 10 mM EDTA, 50 mM Tris-HCl pH 7.4) and 2 μl of Protease Inhibitor Cocktail (Sigma) were added. Co-immunoprecipitation of CsmA-HA, FLAG-ChsB, ΔM-HA, and d10-HA were performed as described previously [25] using the P10 and P100 fractions. mRFP-KinA^rigor^ was detected using a rabbit anti-DsRed-monomer (mDsRed) polyclonal antibody (Cat. No. 632496, Clontech), which recognizes mRFP, at a 1:1,000 dilution and an anti-rabbit immunoglobulin G (IgG), horseradish peroxidase-linked antibody (A0545, Sigma) at a 1:1,000 dilution. Twenty μg proteins were loaded on SDS-PAGE for the detection of proteins tagged with EGFP or mRFP.

## Results

### Localization of ChsB and CsmA

In previous work chitin synthase class III ChsB, expressed from the native promoter and fused to GFP, was observed at hyphal tips and forming septa in *A*. *nidulans* [24]. Here we analyzed the localization mechanism for ChsB and the class V chitin synthase CsmA (S1 Fig). To analyze the dynamic localization of CsmA in living hyphae, we replaced the *csmA* gene with a C-terminally *GFP* tagged version (CsmA-GFP). These strains, however, exhibited phenotypic abnormalities similar to those observed in *csmA*-deletion mutants and only weak fluorescence of CsmA-GFP (data not shown). This suggested that the GFP tag at the C-terminus interferes with the function of CsmA. Thus, we constructed a strain expressing CsmA tagged with GFP at its N-terminus (GFP-CsmA) under the control of the *alcA* promoter (Table 1). This strain grew well and did not exhibit phenotypic abnormalities when the *alcA* promoter was derepressed (medium containing glycerol as a carbon source) or when the *alcA* promoter was induced (medium containing threonine and fructose as carbon sources). The correct insertion of the construct in two strains expressing GFP-CsmA (EGFP-CsmA1 and 2) was confirmed by Southern blot analysis (S2 Fig). The production of the GFP-CsmA protein was confirmed by Western blot analysis using anti-GFP antibodies (S3A Fig).

**Fig 1:**
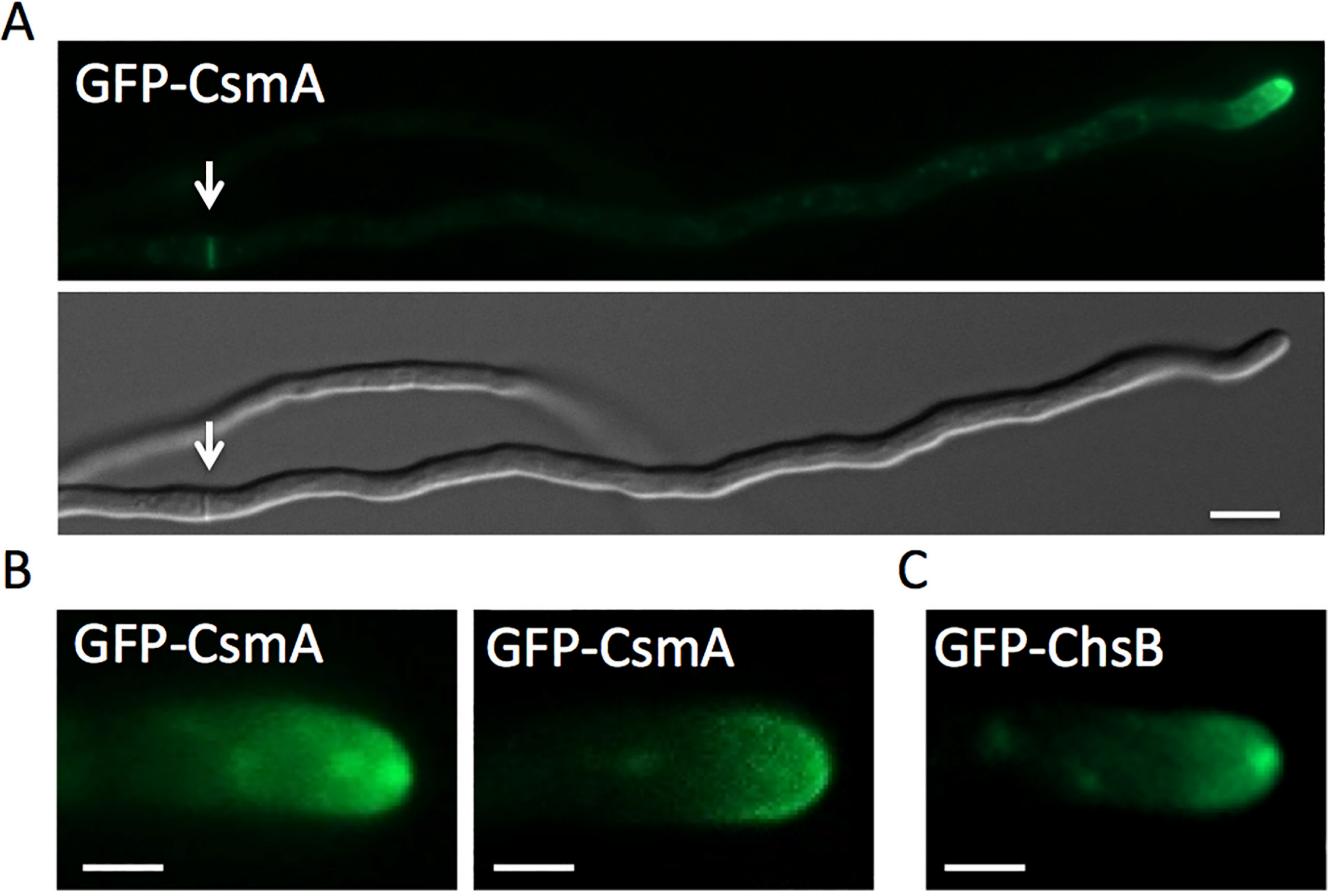
Localization of GFP-CsmA. (A) Strain EGFP-CsmA1 was grown in MMGlyuu overnight. GFP-CsmA was observed at the hyphal tips and forming septa (arrow). (B) GFP-CsmA was found at the Spitzenkörper (left) and/or as a crescent along apical membrane (right). (C) The strain producing GFP-ChsB (EB-5) was grown in MMGlyuu overnight. GFP-ChsB was found mainly at the Spitzenkörper at the hyphal tips. Scale bars represent 5 μm (A) and 2 μm (B,C).

Fluorescence of GFP-CsmA was observed at hyphal tips and at forming septa in addition as moving spots in the cytoplasm (Fig 1A, and data not shown). At the apical membrane, GFP-CsmA was found at the Spitzenkörper and/or as a crescent along the apical membrane (Fig 1B, S1 and S2 Movies). Likewise, GFP-ChsB was observed at hyphal tips and forming septa [24]. However GFP-ChsB was found mainly at the Spitzenkörper at the hyphal tips under this condition (Fig 1C, S3 Movie).

### Role of kinesin-1 (KinA) and kinesin-3 (UncA) for the localization of ChsB and CsmA

To investigate the roles of kinesins, KinA (kinesin-1) and UncA (kinesin-3), for the localization and transportation of ChsB and CsmA, we constructed strains expressing GFP-ChsB or GFP-CsmA in *kinA-* or *uncA*-deletion backgrounds, respectively. The production of GFP-ChsB and GFP-CsmA were confirmed by Western blot analysis using anti-GFP antibodies (S3A and S3B Fig).

**Fig 2:**
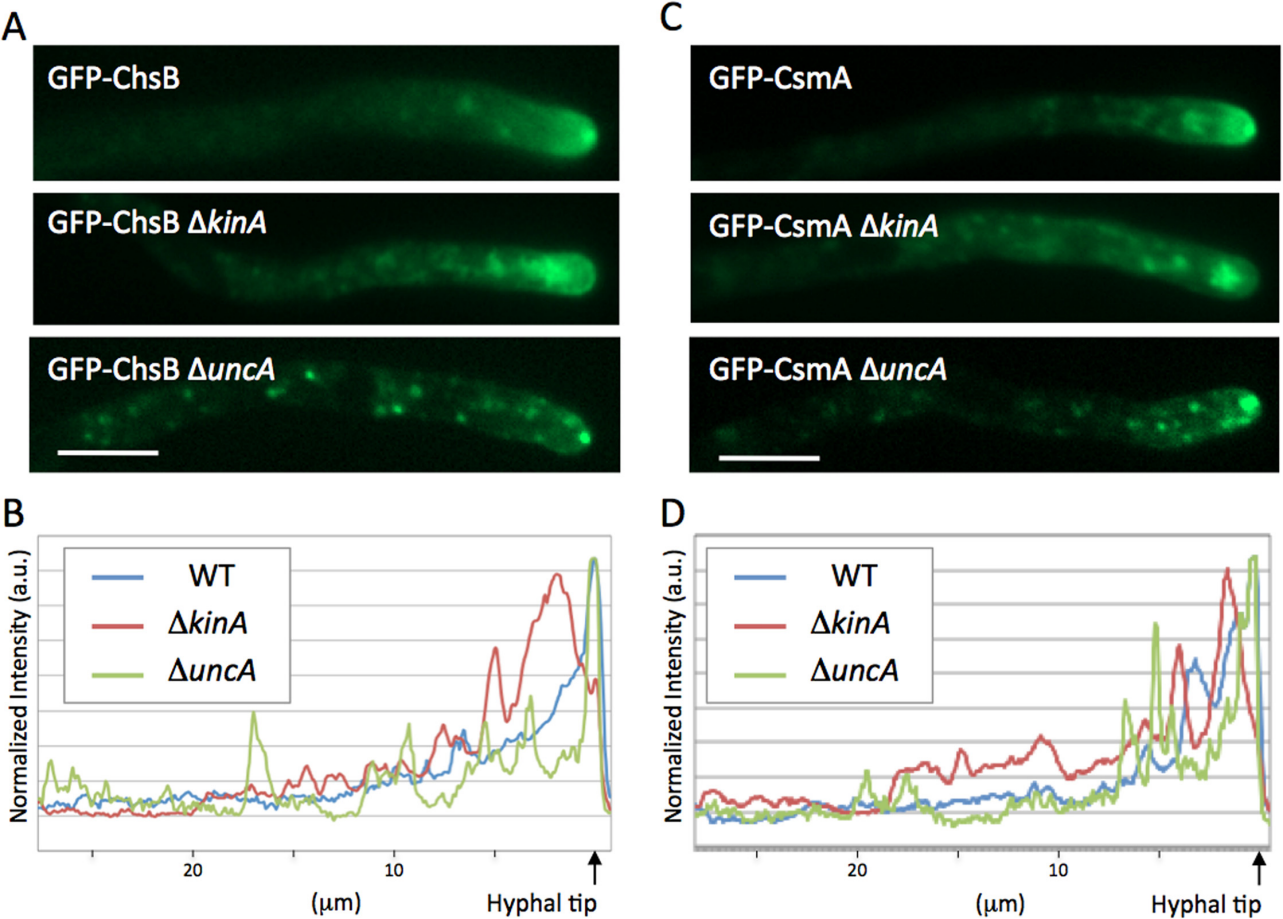
Localization of GFP-ChsB and GFP-CsmA in kinesin-deletion strains. (A, C) The typical localization patterns of GFP-ChsB (A) and GFP-CsmA (C) in wild type (EB-5 and EGFP-CsmA1, respectively), Δ*kinA* (EGFP-ChsBΔkinA1 and EGFP-CsmAΔkinA1, respectively), and Δ*uncA* (EGFP-ChsBΔunkA1 and EGFP-CsmAΔunkA1, respectively) strains grown in MMGlyuu overnight. Scale bars represent 5 μm. (B, D) Signal intensity profiles of GFP-ChsB (B) and GFP-CsmA (D) along hyphae in wild type (blue line), Δ*kinA* (red line), and Δ*uncA* (green line) strains.

Typical localization patterns of GFP-ChsB or GFP-CsmA in the Δ*kinA* or Δ*uncA* are shown in Fig 2A and 2C. In the absence of KinA, both GFP-ChsB and GFP-CsmA showed large accumulations at subapical regions (Fig 2A and 2C). The profiles of GFP signal intensities along hyphae are shown in Fig 2B and 2D. In wild type hyphae, the maximum peaks of GFP-ChsB and GFP-CsmA were restricted sharply at the tip of hyphae. In contrast, the hyphae in *kinA*-deletion strains displayed wider peaks of GFP-ChsB and GFP-CsmA at approximately 1 μm behind the tip of hyphae. In the *uncA*-deletion strains, GFP-ChsB and GFP-CsmA localized at the apex of hyphal tips as in wild type stains (Fig 2A and 2C). However GFP-ChsB and GFP-CsmA appeared to be less diffused behind the apex. In addition, multiple punctate structures were observed along the hyphae. In the profiles of GFP signal intensities, several small peaks were often observed throughout the hyphae in the *uncA*-deletion strains (Fig 2B and 2D).

### Role of KinA and UncA in the transportation of ChsB and CsmA

**Fig 3:**
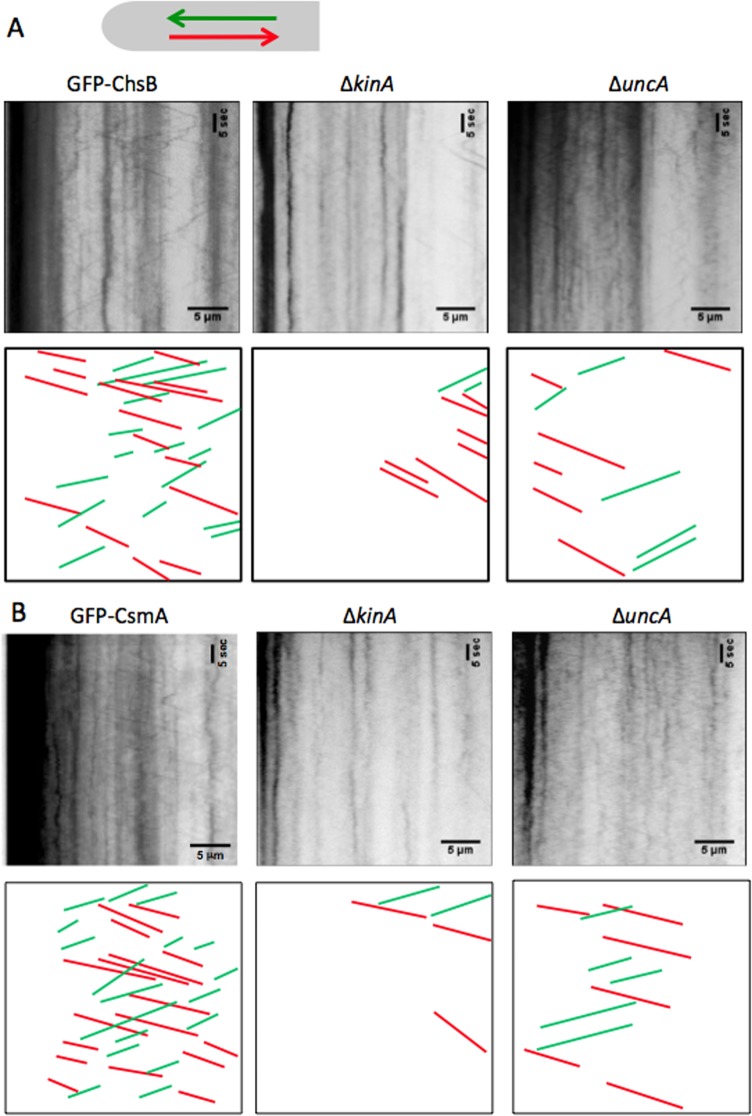
Kymographs GFP-ChsB and GFP-CsmA in kinesin-deletion strains. (A) Kymographs of GFP-ChsB in the wild type, Δ*kinA*, and Δ*uncA* strains. (B) Kymographs of GFP-CsmA in wild type, Δ*kinA*, and Δ*uncA* strains. The kymographs were derived at hyphal tips (30 μm from the tips) from a 1 minute movie (200 ms intervals, total 300 frames). Horizontal scale bars represent 5 μm. Vertical scale bars represent 5 seconds. Anterograde movements are represented by green and retrograde movements are represented by red lines.

We investigated the roles of kinesins, KinA and UncA, in the transportation of ChsB and CsmA. To visualize the transportation events of GFP-ChsB and GFP-CsmA, kymographs were recorded at hyphal tips in wild type, the *kinA*-deletion, and the *uncA*-deletion strains (Fig 3)(S4–S9 Movies). The signals forming vertical lines in the kymographs represent immobile accumulations of GFP-ChsB (Fig 3A) and GFP-CsmA (Fig 3B). With the used microscopy setup it is impossible to detect single GFP molecules. The transport events shown in the kymographs are thought to represent events that multiple GFP-ChsB or GFP-CsmA molecules are transported together. It is not defined here whether the spots represent secretory vesicles, chitosomes, or early endosomes.

### Transport frequency of ChsB and CsmA

**Fig 4:**
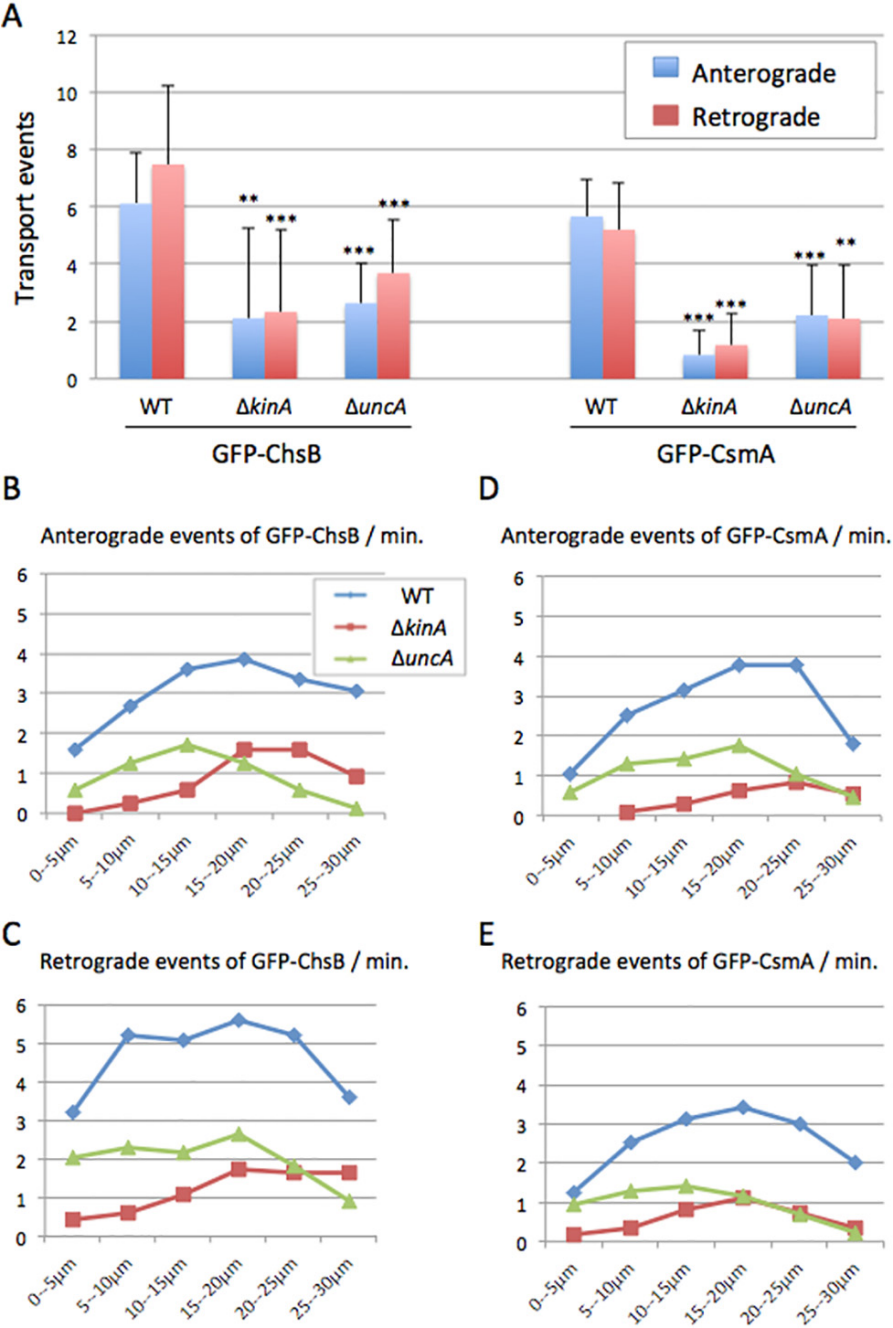
Transport frequency of GFP-ChsB and GFP-CsmA in kinesin-deletion strains. (A) The frequency of anterograde (blue) and retrograde (red) movements of GFP-ChsB (left) and GFP-CsmA (right) in the wild type, Δ*kinA*, and Δ*uncA* strains. Anterograde and retrograde movements were visualized in the kymographs. The numbers of motion events per minute are shown (mean ± S.D). **, P < 0.05 (statistically significant difference); ***, P < 0.001 (highly significant difference), compared to wild type strain. The p values were calculated by unpaired t-test. (B-E) Distribution diagrams of GFP-ChsB anterograde (B) and retrograde (C) events, and GFP-CsmA anterograde (D) and retrograde (E) events in each 5 μm-segment from hyphal tips in the wild type (blue line), Δ*kinA* (red line), and Δ*uncA* (green line) strains.

**Fig 5:**
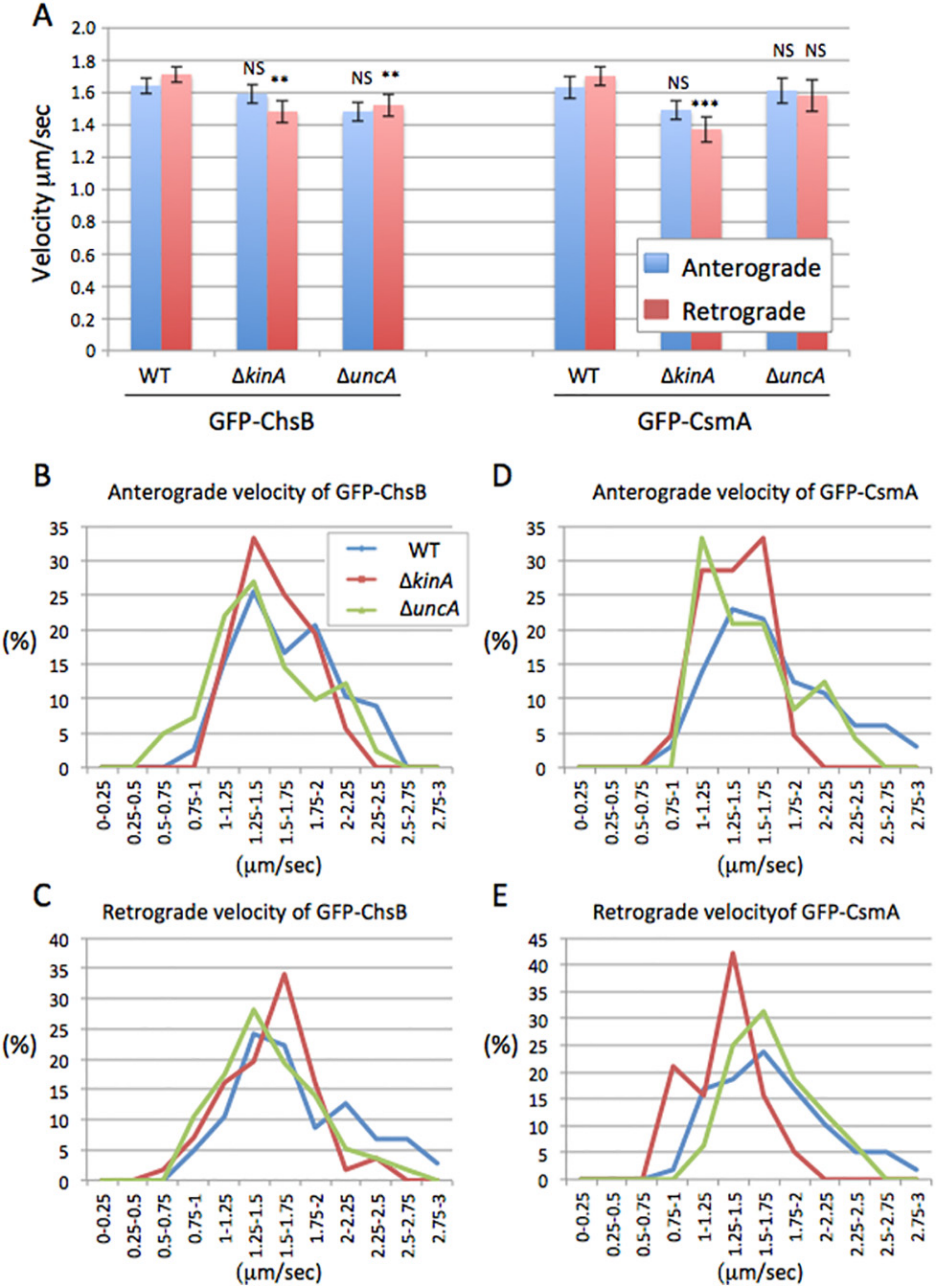
Velocity of GFP-ChsB and GFP-CsmA in kinesin-deletion strains. (A) The velocities of anterograde (blue) and retrograde (red) movements of GFP-ChsB (left) and GFP-CsmA (right) in wild type, Δ*kinA*, and Δ*uncA* strains are shown (mean ± S.E). NS, no significant difference; **, P < 0.05 (statistically significant difference); ***, P < 0.001 (highly significant difference), compared to wild type strain. The p values were calculated by unpaired t-test. (B-E) Distribution of velocities in the GFP-ChsB anterograde (B) and retrograde movements (C) and in the GFP-CsmA anterograde (D) and retrograde movements (E) are shown in wild type (blue line), Δ*kinA* (red line), and Δ*uncA* (green line) strains.

The transport frequency of GFP-ChsB and GFP-CsmA in both anterograde and retrograde direction appeared higher in the wild type than in the *kinA*- or the *uncA*-deletion strains. To compare the transport frequencies, the transport events were quantified from the kymographs (Fig 4). In addition, the velocity of GFP spots was calculated from the kymographs and compared between wild type and the kinesin-deletion strains (Fig 5).

In wild type the frequency of anterograde and retrograde movements visualized by GFP-ChsB were 6.1 ± 1.8 and 7.5 ± 2.7 (mean ± S.D., n = 82 and 100) events per minute, respectively. In the *kinA*-deletion strain those (the frequencies of GFP-ChsB anterograde and retrograde movements) were reduced to 2.1 ± 3.1 and 2.3 ± 2.9 (mean ± S.D., n = 38 and 47) events per minute, respectively. In the *uncA*-deletion strain the frequencies were also reduced to 2.6 ± 1.4 and 3.7 ± 1.9 (mean ± S.D., n = 42 and 58) events per minute, respectively. The frequencies of transports visualized by GFP-ChsB in the *kinA*-deletion and the *uncA*-deletion strains were both significantly lower than those in wild type in anterograde (34% in Δ*kinA* and 46% in Δ*uncA*) and retrograde direction (31% in Δ*kinA* and 48% in Δ*uncA*) (Figs 3 and 4A).

In wild type the frequencies of GFP-CsmA anterograde and retrograde movements were 5.7 ± 1.3 and 5.2 ± 1.6 (mean ± S.D., n = 100 each) events per minute, respectively. In the *kinA*-deletion strain those (the frequencies of GFP-CsmA anterograde and retrograde movements) were significantly reduced to 0.8 ± 0.8 and 1.2 ± 1.1 (mean ± S.D., n = 40 and 45) events per minute, respectively. In the *uncA*-deletion strain the frequencies of GFP-CsmA anterograde and retrograde movement were reduced to 2.2 ± 1.8 and 2.1 ± 1.9 (mean ± S.D., n = 40 and 50) events per minute, respectively. Both frequencies of moving spots visualized by GFP-CsmA in the *kinA*-deletion and the *uncA*-deletion strains were significantly lower than those in wild type in both anterograde (15% in Δ*kinA*, 39% in Δ*uncA*) and retrograde (23% in Δ*kinA*, 40% in Δ*uncA*) direction (Figs 3 and 4A). Generally, the frequencies of GFP-ChsB movement were relatively higher than those of GFP-CsmA. The difference might represent different expression levels of GFP-ChsB and GFP-CsmA.

To analyze the distribution of transport events along the hyphae, the hyphae were divided into 5 μm-segments starting from hyphal tips and the frequencies of the movements of GFP-ChsB and GFP-CsmA were quantified in each segment in each strain (Fig 4B–4E). In cases where movements of GFP-ChsB and GFP-CsmA were observed in longer distances than 5 μm, they were counted in multiple segments. The frequencies of GFP-ChsB and GFP-CsmA in both anterograde and retrograde movements in the *kinA*-deletion strain were lower than those of the wild type strain. Especially in the region close to the tips (0–15 μm), movements of GFP-ChsB and GFP-CsmA were hardly observed in both anterograde and retrograde directions. The frequencies of GFP-ChsB and GFP-CsmA in both anterograde and retrograde direction in the *uncA*-deletion strain were also lower than those of the wild type strain along the hyphae, in contrast, especially behind the tip region (20–30 μm).

### Transport speed of ChsB and CsmA

Although the frequency of GFP-ChsB and GFP-CsmA movements decreased in the *kinA*- and *uncA*-deletion strains, the velocities of the movements were comparable (Fig 5A). In wild type the velocities of GFP-ChsB anterograde and retrograde movements were 1.6 ± 0.1 and 1.7 ± 0.1 (μm/sec., mean ± S.E., n = 82 and 100), respectively. In the *kinA*-deletion strain those were 1.6 ± 0.1 and 1.5 ± 0.1 (μm/sec., mean ± S.E., n = 38 and 47), respectively, and 1.5 ± 0.1 and 1.5 ± 0.1 (μm/sec., mean ± S.E., n = 42 and 58), respectively, in the *uncA*-deletion strain. There were no significant differences in the velocities of anterograde movements in the kinesin-deletion strain, whereas the velocities of retrograde movements were slightly reduced (87% in Δ*kinA*, 89% in Δ*uncA*).

In the wild type strain the velocities of GFP-CsmA anterograde and retrograde movement were 1.6 ± 0.1 and 1.7 ± 0.1 (μm/sec., mean ± S.E., n = 100 each), respectively (Fig 5A). The velocities of GFP-CsmA showed no significant difference compared to the velocities of GFP-ChsB in both anterograde and retrograde movements, respectively. The velocities of anterograde and retrograde movements also showed no significant difference in both GFP-ChsB and GFP-CsmA. In the *kinA*-deletion strain the velocities of GFP-CsmA anterograde and retrograde movement were 1.5 ± 0.1 and 1.4 ± 0.1 (μm/sec., mean ± S.E., n = 40 and 45), respectively. In the *uncA*-deletion strain those were 1.6 ± 0.1 and 1.6 ± 0.1 (μm/sec., mean ± S.E., n = 40 and 50), respectively. There were no significant differences in the velocities of anterograde movements in the kinesin deletion strains, whereas the velocities of retrograde movements were slightly reduced in the *kinA*-deletion strain (81%) but not in the *uncA*-deletion strain.

The distribution of velocities in the anterograde and retrograde movements of GFP-ChsB and GFP-CsmA was analyzed in each strain (Fig 5B–5E). Velocities faster than 2 μm/sec were observed in wild type at all the anterograde and retrograde movements of GFP-ChsB and GFP-CsmA. However, the high speeds were not clearly found in the *kinA*-deletion strain. The average speed of early endosomes transport is slower than that of secretory vesicles (unpublished results). The higher speed peaks might indicate those signals derived from secretory vesicles.

### Transport of ChsB and CsmA to the hyphal tips along microtubules by KinA

**Fig 6:**
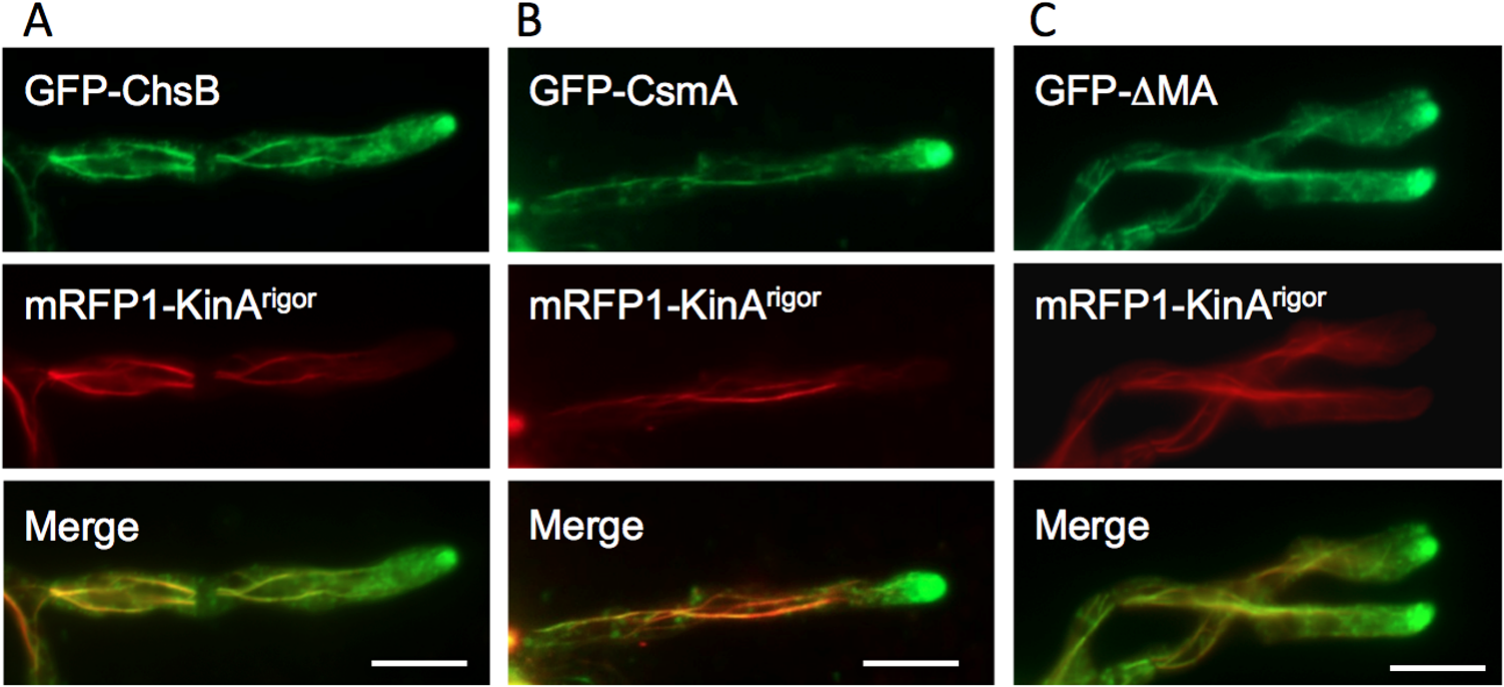
Colocalization of GFP-ChsB, GFP-CsmA, and GFP-ΔMA with mRFP1-KinA^rigor^ along microtubules. (A, B) GFP-ChsB (A) and GFP-CsmA (B) localized to large accumulations at the subapical tips similar to those in the Δ*kinA* strains, and were observed along microtubules decorated with mRFP-KinA^**rigor**^ throughout the hyphae. (C) GFP-ΔMA (CsmA without the MMD) also localized to large accumulations at the subapical tips and along microtubules decorated with mRFP-KinA^**rigor**^. (A-C) These strains were grown in MMGlyuu overnight. Scale bars represent 5 μm.

To further analyze the function of KinA in the transport of GFP-ChsB and GFP-CsmA, we constructed strains co-expressing either GFP-ChsB and mRFP-KinA^rigor^ (EGFP-ChsB/mRFP-KinA^rigor^ strain), or GFP-CsmA and mRFP-KinA^rigor^ (EGFP-CsmA/mRFP-KinA^rigor^ strain). The KinA^rigor^ protein, which has a point mutation in its ATPase domain, binds tightly to microtubules but does not move along them. Consequently, KinA^rigor^ decorates microtubules [48]. GFP-ChsB and GFP-CsmA localized to large accumulations at subapical regions similar to those in *kinA-*deletion strains. In addition, GFP-ChsB and GFP-CsmA were observed along microtubules decorated with mRFP-KinA^rigor^ throughout the hyphae (Fig 6A and 6B). These results suggest that ChsB and CsmA are transported to the hyphal tip regions along microtubules by KinA.

Only the chitin synthase domain of CsmA without the MMD (GFP-ΔMA) did not show a clear accumulation at the hyphal tip membrane (data not shown)[25]. When the GFP-ΔMA was co-expressed with mRFP-KinA^rigor^ in the EGFP-ΔMA/ mRFP-KinA^rigor^1 strain, the GFP-ΔMA also exhibited a similar localization pattern with GFP-CsmA co-expressed with mRFP-KinA^rigor^, along microtubules and at subapical large accumulations (Fig 6C), suggesting that CsmA is transported to the hyphal tip regions by KinA independently of its MMD.

### Interaction of ChsB and CsmA with KinA

**Fig 7:**
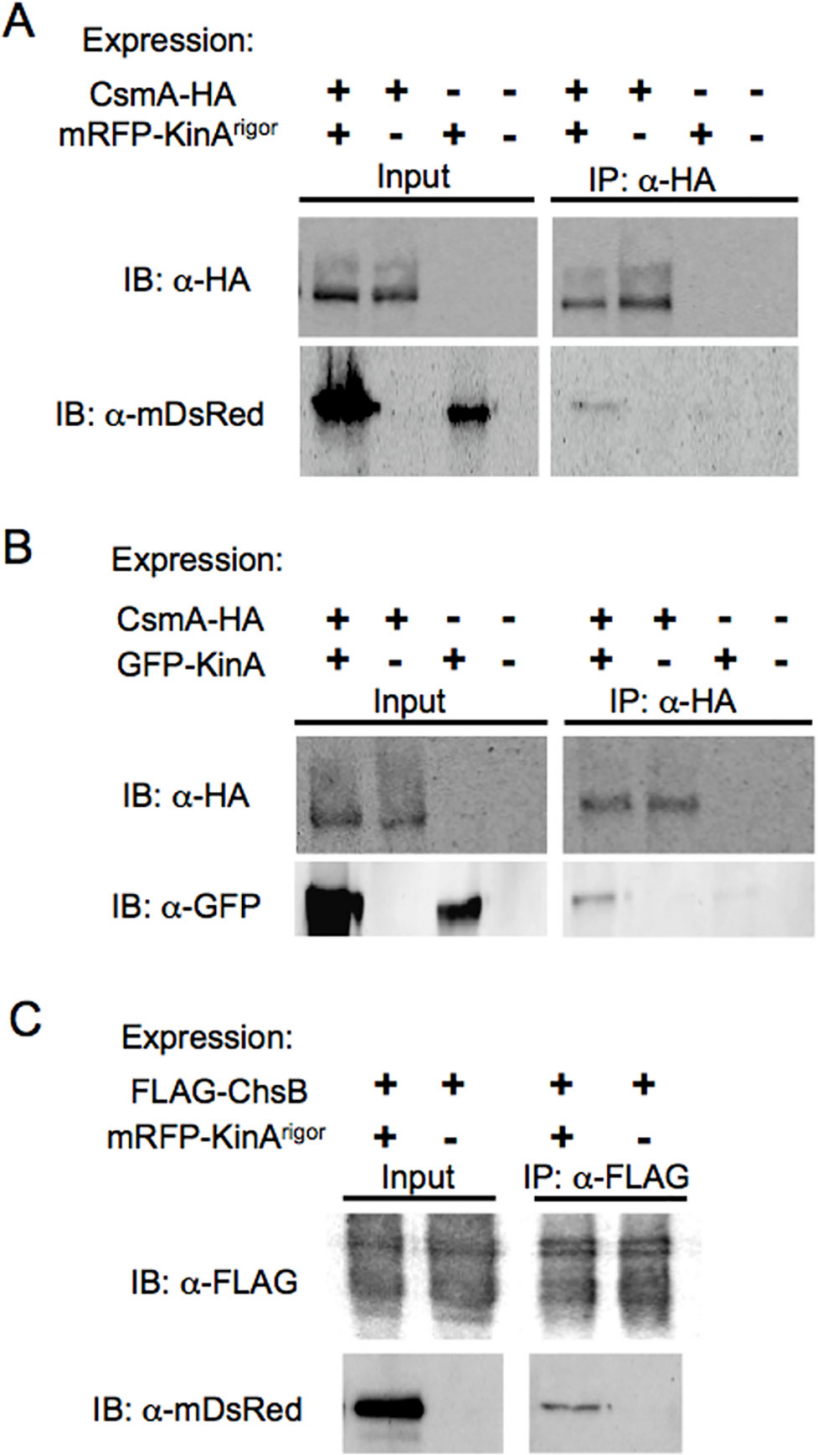
Co-immunoprecipitation of CsmA-HA with mRFP-KinA^rigor^ or GFP-KinA and FLAG-ChsB with mRFP-KinA^rigor^ *in vivo*. (A, B) 100,000 xg pellets (P100) of cell extracts of the strains expressing CsmA-HA, mRFP-KinA^**rigor**^, or both of them (A), and the strains expressing CsmA-HA, GFP-KinA, or both of them (B) were immunoprecipitated with anti-HA antibody. P100 (Input) and immunoprecipitates with anti-HA antibody (IP: α-HA) were subjected to Western blot analysis using antibodies indicated by IB. (C) Those of the strains expressing FLAG-ChsB or FLAG-ChsB and mRFP-KinA^**rigor**^ were immunoprecipitated with anti-FLAG antibody and subjected to Western blot analysis.

To further examine the interaction between ChsB and KinA^rigor^, or CsmA and KinA^rigor^ *in vivo*, we constructed strains co-expressing FLAG-ChsB and mRFP-KinA^rigor^, or CsmA-HA and mRFP-KinA^rigor^ (FLAG-ChsB/mRFP-KinA^rigor^ strain or CsmA-HA/mRFP-KinA^rigor^ strain, respectively). The strains were subjected to a fractionation experiment (see Materials and methods). FLAG-ChsB and CsmA-HA were detected in a 10,000 x g pellet (P10) and in a 100,000 x g pellet (P100) that were obtained by cell fractionation of the crude cell extract of *A*. *nidulans* [24,61]. In *S*. *cerevisiae*, P100 fraction contains transport vesicles, endosomal membranes, and Golgi cisternae [63]. FLAG-ChsB and CsmA-HA were co-immunoprecipitated with mRFP-KinA^rigor^ from the P100 fractions of the FLAG-ChsB/mRFP-KinA^rigor^ strain and CsmA-HA/mRFP-KinA^rigor^ strain, respectively (Fig 7A and 7C). CsmA-HA was also co-immunoprecipitated with wild type KinA from the P100 fraction of the strain expressing CsmA-HA and GFP-KinA (Fig 7B). These results suggest that transport vesicles containing ChsB or CsmA are transported through the interaction with KinA. In addition, CsmA without the MMD (ΔMA) and CsmA truncated mutant protein lacking 10 amino acids from the actin binding domain of the MMD (D10M) also co-immunoprecipitated with mRFP-KinA^rigor^ (S4 Fig)[25], suggesting that the interaction of transport vesicles containing CsmA with KinA is independent of the MMD.

We examined the interaction between ChsB and CsmA, but we did not detect co-immunoprecipitation of CsmA-HA or FLAG-ChsB from the P100 fractions of the strains that produce these tagged proteins together with or without mRFP-KinA^rigor^ (FLAG-ChsB/CsmA-HA/mRFP-KinA^rigor^ strain or FLAG-ChsB/CsmA-HA strain, respectively) (data not shown). These results suggest that ChsB and CsmA are transported on different vesicles by KinA.

## Discussion

In filamentous fungi, protein machineries required for polarized growth, such as formin and regulators of polarity establishment, accumulate in the Spitzenkörper at the hyphal tips [3,5,7,10,12]. Chitin synthases of the classes I, III, and VII of *Neurospora crassa* (Chs3, Chs1, and Chs6) also localized at the Spitzenkörper and in subapical regions as punctate structures [55,64]. In the previous studies, we investigated the localization of class III and V chitin synthases in *A*. *nidulans*, ChsB and CsmA, respectively [24,25]. In this study, we investigated the transport mechanisms of ChsB and CsmA in living hyphae, especially the roles of kinesin-1 (KinA) and kinesin-3 (UncA) in the transportation of ChsB and CsmA. Our results suggest that these chitin synthases are delivered to hyphal tips along microtubules through the interaction with conventional kinesin (kinesin-1, KinA).

The localizations of GFP-CsmA and GFP-ChsB were disturbed especially around the hyphal tip region by the *kinA* deletion (Figs 2–5). In addition these chitin synthases co-immunoprecipitated with KinA. These results suggest that KinA transports CsmA and ChsB. Movements of ChsB and CsmA were hardly observed in *kinA*-deletion mutants and the number of movements with higher velocities were reduced in the mutants. Since it is suggested that smaller vesicles tend to move faster than larger vesicles [65], it is possible that these chitin synthases are mainly transported on small vesicles, such as secretory vesicles or chitosomes, at extreme hyphal tip regions by KinA. However, KinA is required for the transport of cytoplasmic dynein to the microtubule plus ends [52] and dynein itself is involved in membrane transport such as early endosomes in several fungi [11,47,66,67]. Since ChsB and CsmA accumulated at subapical regions of the hyphae in the *kinA*-deletion mutant and dynein is not transported to those regions in the *kinA*-deletion mutants [52], it is also possible that the defects of ChsB and CsmA transport in the *kinA*-deletion strains include the direct and/or indirect effect of mislocalization of dynein.

In *U*. *maydis*, Schuster et al. reported that class V chitin synthase Mcs1 was also transported by kinesin-1 [50]. These results indicate that the transport mechanism of class V chitin synthases is conserved between ascomycete and basidiomycete fungi. In contrast, the transport of the class III chitin synthase in *N*. *crassa* does not depend strictly on microtubules [64], suggesting that the transport mechanism of class III chitin synthase of *A*. *nidulans* is at least partially different from that of another ascomycete *N*. *crassa*. Since CsmA-HA and FLAG-ChsB did not co-immunoprecipitate, they are likely transported in different vesicle populations. In *N*. *crassa*, it is also suggested that classes I, III, and VII chitin synthases are contained in different vesicular subpopulations [64]. Thus chitin synthases of different classes are possibly transported in different vesicle subpopulations.

Kinesin-3, UncA, is involved in the transport of early endosome and peroxisome [11,48,68]. In *U*. *maydis*, kinesin-3 is also involved in the transport of early endosomes and the secretion of acid phosphatase [46,67,69], but is not involved in the transport of Mcs1 [50]. Although the transports of GFP-ChsB and GFP-CsmA were disturbed to some extent in the *uncA*-deletion strain, the localization at hyphal tips appeared nearly normal in the mutants. This suggests that UncA is involved in the transport of ChsB and CsmA, but not essential especially in hyphal tip regions. In *A*. *nidulans*, actin forms a ring structure at the subapical plasma membrane [70]. Endocytosis is suggested to occur nearby this structure, and the resultant endocytic vesicles fuse with each other in the cytoplasm to form early endosomes [7,70–73]. The early endosomes are transported along microtubules by kinesin-3 and dynein in the plus and minus end directions, respectively, in hyphae of *A*. *nidulans* and *U*. *maydis* [6,11,46,48,49] and the endosome system is thought to be involved in the recycling of membrane proteins at hyphal tips [74]. It is possible that kinesin-3, UncA, is involved in the transport of ChsB and CsmA through the recycling pathway. Alternatively the defects of early endosome transport might affect the transport of ChsB and CsmA indirectly. It is necessary to distinguish secretory vesicles and early endosomes in same hyphae in order to investigate the dependency of KinA and UncA on the transport.

It is well known that actin is also crucial for hyphal tip growth and that myosins are motor proteins that move on actin filaments. *A*. *nidulans* contains three genes, *myoA*, *myoB*, and *myoE* that encode myosin heavy chains belonging to the myosin families, myosin-1, myosin-2, and myosin-5, respectively [8,9,75]. Moreover, MyoE participates in vesicle transport in hyphae [8]. Mutagenesis of both, myosin-5 and kinesin-1 caused severe growth defects in *A*. *nidulans* and *U*. *maydis* [9,69], suggesting that the function of kinesin-1 could be partly substituted by myosin-5 and *vice versa*. Since myosin-5 functions in the transport of Mcs1 to the cell surface in a microtubule-independent manner [50], it is possible that a myosin-5, MyoE, is also involved in the transport of CsmA and ChsB. This idea is supported by the fact that the inhibition of actin polymerization by cytochalasin A severely disturbed the localization of these chitin synthases (data not shown)[25]. The MMDs of class V chitin synthases belong to the myosin-17 family. The binding of the MMD to actin filaments is required for the proper localization and function of CsmA [25], whereas no motor activity of the MMD of Mcs1 was detected *in vitro* [50]. The point mutation at the P-loop or Switch I in the MMD of CsmA does not affect its function and localization [25], suggesting that there is no motor activity or at least the motor activity is not essential for the transport mechanism. The idea is supported by our results that CsmA and CsmA without the MMD is transported to the hyphal tips by KinA.

If the MMD of CsmA is not necessary for the transport to hyphal tips, the role of MMD remains unrevealed. Schuster et al. suggested that the MMD of Mcs1 functions in the tethering of vesicles containing Mcs1 near the sites of exocytosis [50]. Our results showed that the deletion of kinesin-1 or kinesin-3 caused similar effects on the transport of ChsB and CsmA. The role of MMD may be important for the localization at hyphal tips after their transportation, rather than the transport towards hyphal tips. GFP-CsmA was found at the Spitzenkörper and/or as a crescent along the apical membrane (Fig 1B). In contrast, GFP-ChsB was found mainly at the Spitzenkörper at hyphal tips (Fig 1C). The difference in the localization between CsmA and ChsB was also observed in a significant population of hyphal tips by indirect immunofluorescence analysis [24,25]. ChsB and CsmA are likely to be transported to the Spitzenkörper in a similar way. Then, CsmA might be localized and/ or stay at special regions along the apical membrane depending on the interaction of MMD with the actin cytoskeleton.

## Supporting Information

S1 Fig Phylogenetic tree of fungal chitin synthases. Alignment was done with the clustalW alignment program using the entire amino acid sequence of each chitin synthase. The tree was drawn using Njplot. Branch length indicates evolutionary distance. Chitin synthases belong to class III and V are shown in red and blue, respectively. Abbreviations: Nc, *N*. *crassa*; Sc, *S*. *cerevisiae*; Um, *U*. *maydis*.
(TIF)

S2 Fig Construction of GFP-CsmA producing strains. (A) Strategy for constructing GFP-CsmA producing strains. (B) Southern blot analysis of *Eco*RV digested total DNAs of the EGFP-CsmA strains using the 1.0 kb *Spe*I-*Xho*I fragment of pM-ALK-CHS5 [23] as a probe. Lane 1, ABPU1; lane 2, EGFP-CsmA1; lane 3, EGFP-CsmA2.
(TIF)

S3 Fig Western blot analysis of the cell lysates of EGFP-CsmA or EGFP-ChsB producing strains. (A) GFP-CsmA in the cell lysates of the EGFP-CsmA1 (WT), EGFP-CsmA/ΔkinA1 strain (Δ*kinA*), and EGFP-CsmA/ΔuncA1 (Δ*uncA*) strains were detected with anti-GFP antibody. (B) EGFP-ChsB in the cell lysates of the EB-5 strain (WT), EGFP-ChsB/ΔkinA1 (Δ*kinA*), and EGFP-ChsB/ΔuncA1 (Δ*uncA*) strains were detected using the same antibody. P10, 10,000 x g pellet; P100, 100,000 x g pellet; S100, 100,000 x g supernatant.
(TIF)

S4 Fig Co-immunoprecipitation of ΔMA-HA with mRFP-KinA^rigor^ and D10M-HA with mRFP-KinA^rigor^. P100 fractions of the CΔMHA strain or the CΔMHA/mRFP-KinA^rigor^1 strain (A), and the D10HA strain or the D10HA/mRFP-KinA^rigor^1 strain (B) were immunoprecipitated with anti-HA antibody and subjected to Western blot analysis.
(TIF)

S1 Movie GFP-CsmA localized at Spitzenkörper (400 ms interval, total 30 seconds, Scale bar 2 μm). (AVI)

S2 Movie GFP-CsmA localized as a crescent along apical membrane (500 ms interval, total 28 seconds, Scale bar 2 μm). (AVI)

S3 Movie GFP-ChsB localized at Spitzenkörper (200 ms interval, total 30 seconds, Scale bar 2 μm). (AVI)

S4 Movie GFP-ChsB in wild type strain (200 ms interval, total 60 seconds, Scale bar 5 μm). (AVI)

S5 Movie GFP-CsmA in wild type strain (200 ms interval, total 60 seconds, Scale bar 5 μm). (AVI)

S6 Movie GFP-ChsB in Δ*kinA* strain (200 ms interval, total 60 seconds, Scale bar 5 μm). (AVI)

S7 Movie GFP-CsmA in Δ*kinA* strain (500 ms interval, total 60 seconds, Scale bar 5 μm). (AVI)

S8 Movie GFP-ChsB in Δ*uncA* strain (200 ms interval, total 60 seconds, Scale bar 5 μm). (AVI)

S9 Movie GFP-CsmA in Δ*uncA* strain (200 ms interval, total 60 seconds, Scale bar 5 μm). (AVI)
