# Supplementary figures and images for: Transportation of Aspergillus nidulans Class III and V Chitin Synthases to the Hyphal Tips Depends on Conventional Kinesin
Source: PLoS One. 2015 May 8;10(5):e0125937. doi: 10.1371/journal.pone.0125937 (PMC4425547; doi:10.1371/journal.pone.0125937)

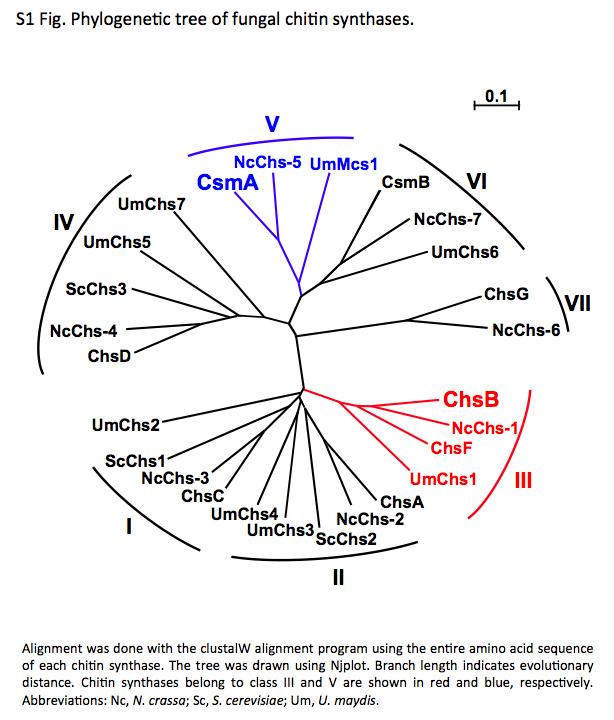

Supplement: S1 Fig — Alignment was done with the clustalW alignment program using the entire amino acid sequence of each chitin synthase. The tree was drawn using Njplot. Branch length indicates evolutionary distance. Chitin synthases belong to class III and V are shown in red and blue, respectively. Abbreviations: Nc, N. crassa; Sc, S. cerevisiae; Um, U. maydis. (TIF) [file pone.0125937.s001.tif]

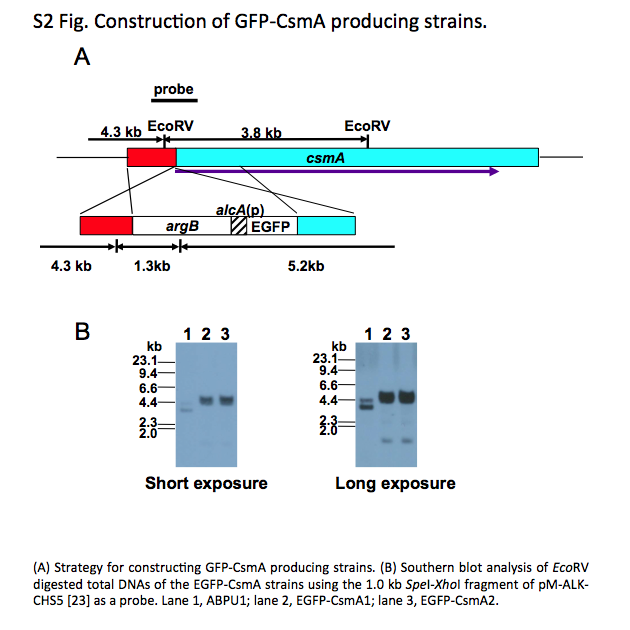

Supplement: S2 Fig — (A) Strategy for constructing GFP-CsmA producing strains. (B) Southern blot analysis of EcoRV digested total DNAs of the EGFP-CsmA strains using the 1.0 kb SpeI-XhoI fragment of pM-ALK-CHS5 [23] as a probe. Lane 1, ABPU1; lane 2, EGFP-CsmA1; lane 3, EGFP-CsmA2. (TIF) [file pone.0125937.s002.tif]

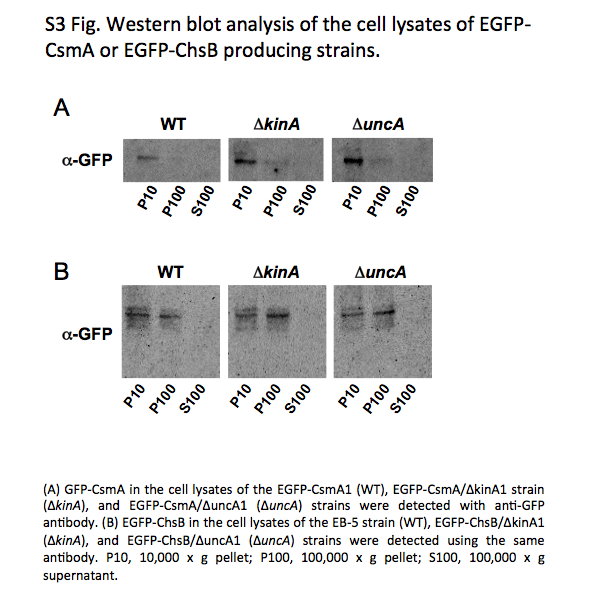

Supplement: S3 Fig — (A) GFP-CsmA in the cell lysates of the EGFP-CsmA1 (WT), EGFP-CsmA/ΔkinA1 strain (ΔkinA), and EGFP-CsmA/ΔuncA1 (ΔuncA) strains were detected with anti-GFP antibody. (B) EGFP-ChsB in the cell lysates of the EB-5 strain (WT), EGFP-ChsB/ΔkinA1 (ΔkinA), and EGFP-ChsB/ΔuncA1 (ΔuncA) strains were detected using the same antibody. P10, 10,000 x g pellet; P100, 100,000 x g pellet; S100, 100,000 x g supernatant. (TIF) [file pone.0125937.s003.tif]

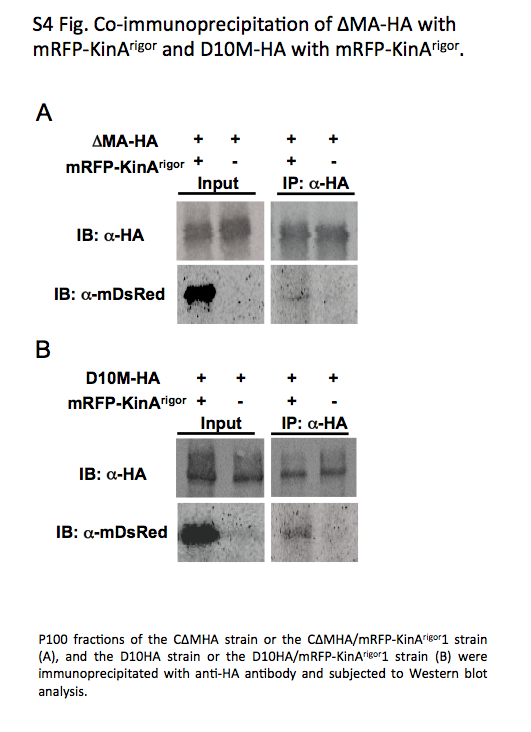

Supplement: S4 Fig — P100 fractions of the CΔMHA strain or the CΔMHA/mRFP-KinArigor1 strain (A), and the D10HA strain or the D10HA/mRFP-KinArigor1 strain (B) were immunoprecipitated with anti-HA antibody and subjected to Western blot analysis. (TIF) [file pone.0125937.s004.tif]
